# Experiences of research ethics committee members and scientists of the research protocol review process in Uganda: a case study

**DOI:** 10.1093/inthealth/ihaa047

**Published:** 2020-11-09

**Authors:** Agnes Ssali, Fiona Poland, Janet Seeley

**Affiliations:** MRC/UVRI & LSHTM Uganda Research Unit, P.O. Box 49, Entebbe; University of East Anglia, Norwich UK Norwich Research Park, Norwich NR4 7TJ, UK; MRC/UVRI & LSHTM Uganda Research Unit, P.O. Box 49, Entebbe; London School of Hygiene and Tropical Medicine, 15–17 Tavistock Place, London WC1H 7SH, UK

**Keywords:** ethics committee, process, protocol, research, review, Uganda

## Abstract

**Background:**

We investigated how relevant and responsive scientists and research ethics committee (REC) members considered the research protocol review processes for health research practice in Uganda.

**Methods:**

Interviews were conducted with five scientists and five REC members. Data were analysed thematically.

**Results:**

How much to compensate for time, the amount of study information shared with volunteers and sample storage for future unknown research were areas of concern for REC members. Delays in getting feedback concerned scientists.

**Conclusions:**

Researchers and REC members need to hold regular discussions to ensure the review process is relevant and responsive.

## Introduction

Scientists and research ethics committee (REC) members have expressed concerns that the review process does not factor in certain aspects of trial conduct among vulnerable populations, such as compensation for time, amount of study information and future sample storage. This may lead to some friction between the two groups if not discussed.^1^ We investigated how the ethics review process might become more relevant and responsive to health research practice in Uganda and in the wider field of applied research ethics.

## Methods

Following ethical approval for the study, we conducted in-depth interviews with five Uganda Virus Research Institute (UVRI) REC members of different disciplines: statistician, public health specialist, social scientist, clinician and a community representative and five scientists involved in human immunodeficiency virus (HIV) clinical trials with experience submitting protocols for review. Study information was shared and written informed consent was obtained. A semi-structured interview topic guide was used for individual interviews. Interviews were digitally recorded after participants had given their consent for recording. Data analysis was thematic following the patterns coming through the data; the process of analysis was managed using NVivo (QSR International, Melbourne, VC, Australia).

## Results

The REC members described the review process as ‘formal’. Each committee member was tasked with reviewing the whole protocol, although a few members with expertise in the given study topic area and discipline would take the lead in the discussion. REC members argued that scientists should facilitate the review process by ensuring their applications are clear, clarifying concepts and shortening the participant information documents.

REC members reported a number of challenges: ensuring information sheets and consent forms were clear and could be understood by the targeted study populations; decisions about appropriate compensation to study participants; researcher requests for sample storage without clear reasons for the purpose being explained; managing the response from scientists who disputed the REC feedback on reviewed protocols; funding limits in the field of ethics, which meant the training of committees and researchers in research ethics was limited; and the absence of national policy guidelines on research ethics. The issue of offering compensation, especially in poor communities or even among vulnerable participants, is a design decision the REC members had to discuss and was noted as ‘difficult’.

The REC members noted that while not a common practice, there were occasions when they requested a change to certain protocols and the collaborating partners working with UVRI researchers undermined their suggestions, going to a different review board for a more favourable ethics review and then proceeding with the study. This threatened ethical standards and underlined the importance of shared standards followed by different RECs in Uganda.

The review process was seen by the scientists as a very formal legalistic process. They reported that they regularly had to wait for a long time for approval before they could start their research. The challenges reported by scientists included waiting for a long time before receiving approval for a study, lack of a formal curriculum to train their research teams, difficulty in assessing with confidence whether a participant had understood the study information and having to communicate scientific terms in the informed consent documents. It was noted that comprehension of the study purpose and procedures was not dependent on literacy skills; attention to ensuring the process of obtaining consent was clear and understandable was essential for all.^2^ However, the amount of information required in consent documents (sometimes required by funders and study sponsors) remained a challenge. There continues to be discussion over the content and length of such documents.^3^

## Discussion

Conducting HIV clinical trials raises ethical and scientific design issues that may impact on the ethics review process. A therapeutic misconception, where a volunteer agrees to take part in research as a surrogate for health service provision that may not normally be available where health services are limited, was of concern to both researchers and REC members.

Approving storage of samples on the basis of ‘broad consent’ without the researchers stating clearly what the samples would be used for, is a critical area for review, requiring the REC members to weigh the costs and benefits of the research for study volunteers and question whether, if they approved, they are ‘doing the right thing’.^4^ The development of a guiding framework on these aspects of ethical conduct to be used across all RECs would assist in this decision making.

## Conclusions

The practical experiences shared by participants in this study can help improve communication between RECs and researchers by highlighting debates and dilemmas raised by REC members in handling the ethics review process. The protocol review process is evolving and there is a need for researchers and REC members to hold regular discussions to ensure the review process is relevant and responsive to health research practice in a given research context.
